# A Novel Wide-Area Backup Protection Based on Fault Component Current Distribution and Improved Evidence Theory

**DOI:** 10.1155/2014/493739

**Published:** 2014-06-19

**Authors:** Zhe Zhang, Xiangping Kong, Xianggen Yin, Zengli Yang, Lijun Wang

**Affiliations:** ^1^State Key Laboratory of Advanced Electromagnetic Engineering and Technology, Huazhong University of Science and Technology, 1037 Luoyu Road, Wuhan 430074, China; ^2^State Grid Jiangsu Electric Power Company, Electric Power Research Institute, 1 Power Avenue, Nanjing 211103, China; ^3^State Grid Hubei Electric Power Company, 175 Xudong Street, Wuhan 430074, China

## Abstract

In order to solve the problems of the existing wide-area backup protection (WABP) algorithms, the paper proposes a novel WABP algorithm based on the distribution characteristics of fault component current and improved Dempster/Shafer (D-S) evidence theory. When a fault occurs, slave substations transmit to master station the amplitudes of fault component currents of transmission lines which are the closest to fault element. Then master substation identifies suspicious faulty lines according to the distribution characteristics of fault component current. After that, the master substation will identify the actual faulty line with improved D-S evidence theory based on the action states of traditional protections and direction components of these suspicious faulty lines. The simulation examples based on IEEE 10-generator-39-bus system show that the proposed WABP algorithm has an excellent performance. The algorithm has low requirement of sampling synchronization, small wide-area communication flow, and high fault tolerance.

## 1. Introduction

With the development of communication technology and the popularity of power communication network based on synchronous digital hierarchy (SDH) fiber ring network, the study of wide-area backup protection (WABP) based on fault element identification has attracted great attention [1–8]. WABP has become one of the most important development directions of relaying protection in the context of smart grid.

An optimization strategy for the fault diagnosis in power systems based on genetic algorithm-Tabu search (GATS) is proposed in [1]. In the fault diagnosis strategy, action states of protections and breaker statuses are adopted as information sources. Current differential backup protection for a busbar and transmission circuits connected to that busbar which is on an interstation or wide-area basis is proposed in [2]. Wide-area backup protection algorithm based on the fault component voltage distribution is proposed in [3]. A wide-area backup protection proposed in [4] is based on phasor information of voltage and current. A PMU-based fault location method based on voltage measurements is developed in [5]. Wide-area backup protection expert systems presented in [6, 7] take action states of four-zone distance relays as information sources. A wide-area relaying protection algorithm based on longitudinal comparison principle is proposed in [8]. Generally speaking, the existing WABP algorithms can be mainly classified into 2 kinds: *①* WABP based on multiend electrical quantity information; *②* WABP based on logic quantity information. For the existing WABP algorithms, there are three main problems.

1. For the WABP algorithms based on multiend electrical quantity information, like wide-area current differential protection [2], the requirement of sampling synchronization is so high that it is difficult to be realized in practice. Synchronization technique based on GPS or Compass has high synchronization accuracy which is the most suitable way for WABP, but the radio communication way of GPS or Compass has the problem of interference deception and other hidden risks. Therefore, in the electric power system in China, relay protection could not rely on any external clock source. In [9], a new current differential protection based on nonlinear restraint criterion is proposed, and the tolerance of synchronization error could reach 3 ms. However, the sampling synchronization between substations based on WAMS cannot effectively keep synchronization error within 3 ms, since the WAMS is a software real-time communication network [10] without external clock source.
2. For the existing WABP algorithms, the requirements of wide-area communication network and processing performance of decision-making center are strict, since too much information is uploaded to the decision-making center. In [2, 3], it proposes that the information of transmission line is uploaded to master substation only if the corresponding starting element acts. Under this condition, the amount of communication flow is reduced. However, the action domain of starting element is so large that the reduction is not significant. Meanwhile, large amount of information presents a severe challenge to the processing performance of the decision-making center. Hence, how to reduce the communication flow and processing burden of the decision-making center is an important issue to be solved for the existing WABP algorithms.
3. The fault tolerance of the existing WABP algorithms needs to be further improved. WABP needs to collect multipoint measurement information of power grid to identify fault element. It is difficult to avoid the mistake or loss of information caused by equipment failure or damage of communication network. It will even face the serious situation that all information of a substation is lost due to failure of DC power supply. However, the performances of the existing WABP algorithms will be poor if relevant information is mistaken or lost.

In order to solve the above three problems of the existing WABP algorithms, this paper proposes a novel WABP based on the distribution characteristics of fault component current and improved Dempster/Shafer (D-S) evidence theory.  Section 2 analyzes the distribution characteristics of fault component current when a fault occurs. An improved D-S evidence theory is proposed in Section 3.  Section 4 introduces the implementation of the proposed WABP. Simulation cases are studied with PSCAD/EMTDC in Section 5 to validate the performance of the proposed WABP.

## 2. Distribution Characteristics of Fault Component Current 

The electrical quantities measured at different positions of power grid are much different from each other and can reflect the fault state of the power grid accurately and timely. The proposed WABP algorithm is based on the distribution characteristics of fault component current to reduce the wide-area communication flow significantly.

**Figure 1:**
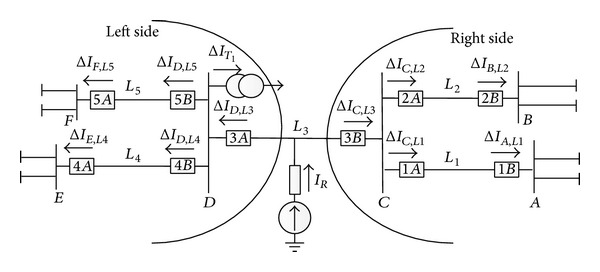
Distribution of fault component currents.

If a fault occurs on *L*_3_, the distribution of fault component current is shown in Figure 1.

### 2.1. The Largest Fault Component Current Will Be Measured at One Terminal of the Actual Faulty Line

When a fault occurs, the amplitude of fault component current gradually increases with the decrease of the distance between the transmission line and the fault point. As shown in Figure 1, due to the current diversion by *L*_1_ and *L*_2_, Δ*I*_*C*,*L*3_ > (Δ*I*_*C*,*L*1_, Δ*I*_*C*,*L*2_). Likewise, for substation *D*, Δ*I*_*D*,*L*3_ > (Δ*I*_*D*,*L*4_, Δ*I*_*D*,*L*5_, Δ*I*_*T*1_). The relative relationship between Δ*I*_*C*,*L*3_ and Δ*I*_*D*,*L*3_ depends on the fault point, line impedance, and system impedance. Anyhow, the larger one of Δ*I*_*C*,*L*3_ and Δ*I*_*D*,*L*3_ is the largest one of the fault component currents in the whole grid.

In actual EHV/UHV power grid, the current diversion is very significant because there are usually many lines connected to one substation. Through the comparison of fault component currents of all transmission lines in the whole gird, only one transmission line which has the maximum fault component current can be accurately identified as the actual faulty line. However, in some extreme cases that the number of connected branches is small or just 2, the current diversion by connected branches will be no longer significant. Under this condition, the fault component current with maximum amplitude may not only be measured at the terminal of the actual faulty line, but also be measured at the terminals of other nonfaulty lines.

As shown in Figure 1, assume that *L*_1_ is out of service or *L*_1_ actually does not exist. In this sense, *L*_3_ and *L*_2_ become series branches. If a fault occurs on *L*_3_, Δ*I*_*C*,*L*2_ will be nearly equal to Δ*I*_*C*,*L*3_ since there are no other shunting branches. Under this condition, if Δ*I*_*D*,*L*3_ > Δ*I*_*C*,*L*3_, the actual faulty line *L*_3_ will have the maximum fault component current. However, if Δ*I*_*D*,*L*3_ < Δ*I*_*C*,*L*3_, two transmission lines which are nonfaulty line *L*_2_ and faulty line *L*_3_, respectively, will have the maximum fault component current.

However, the series branches and other extreme situations are few in actual EHV/UHV power grid. Thus, it is able to identify the actual faulty line under most conditions through the comparison of fault component currents.

### 2.2. In a Single Substation, the Transmission Line Which Is the Closest to the Fault Point Has the Maximum Fault Component Current

As shown in Figure 1, for a single substation, due to the current diversion by nonfaulty lines, fault component current of the transmission line which is the closest to the fault point has the maximum amplitude, like Δ*I*_*C*,*L*3_ > (Δ*I*_*C*,*L*1_, Δ*I*_*C*,*L*2_). Therefore, with the comparison of fault component currents of all connected branches in the local substation, it can identify the transmission line which is the closest to the fault point. Taking substations* A*~*F*, for example, transmission lines *L*_1_, *L*_2_, *L*_3_, *L*_4_, and *L*_5_ can be identified, respectively. Usually, only one transmission line which is the closest to the fault point can be identified for each substation. However, if there are series branches or double circuit lines in power grid, the number of identified transmission lines will be more than one.

Based on the above distribution characteristics, the fault component currents can be used for identification of candidate suspicious faulty line (to be discussed in Section 4.2). In order to meet the requirement of WABP algorithm, the used fault component currents should exist stably for a long time. Hence, if an asymmetrical fault occurs, the negative- or zero-sequence fault current is used for identification of candidate suspicious faulty line. When a symmetrical fault occurs, the positive-sequence fault current, that is, fault current, is used to identify faulty line, considering that the fault current is approximately equal to the fault component current under this condition.

## 3. Improved D-S Evidence Theory

In order to improve the fault tolerance of the proposed WABP algorithm on condition that mistake or loss of information occurs, D-S evidence theory can be adopted. D-S evidence theory [11] can better grasp the uncertainty of problem than traditional probability theories, and it can well deal with the contradiction among multisource information.

### 3.1. Improved D-S Evidence Theory

Suppose that Θ is discernment frame. In this paper, Θ has only two elements which are *A*_1_ representing no fault occurrence on line and *A*_2_ representing fault occurrence on line. If function *m*:2^Θ^ → [0,1] (2^Θ^ = {*∅*, *A*_1_, *A*_2_, *A*_1_ ∪ *A*_2_} which is the power set of Θ) matches the condition

(1) $$\begin{array}{c} m\left(\emptyset \right)=0,\;\;\underset{A\subseteq \Theta ,A\neq \emptyset }{\sum }m\left(A\right)=1, \end{array}$$

*m*  is the basic probability assignment (BPA) on Θ. If *m*(*A*) > 0, *A* is called “focal element.” Assume there are *n* BPAs denoted by *m*_1_, *m*_2_, …, *m*_*n*_. They can be combined to yield a new BPA *m* by Dempster's rule of combination (noted as *m*_1_ ⊕ *m*_2_ ⊕ ⋯⊕*m*_2_). *m* is defined as

(2) $$\begin{array}{c} m\left(A\right)=\{\begin{array}{cc} \frac{\sum_{\cap A_{i}=A}\;\prod_{1\leq j\leq n}m_{j}\left(A_{i}\right)}{1-K} & A\neq \emptyset \\ 0 & A=\emptyset , \end{array} \end{array}$$

with *K* = ∑_∩*A*_*i*_=*∅*_∏_1≤*j*≤*n*_ *m*_*j*_(*A*_*i*_) which is normalization constant and called “conflict index.” *K* measures the conflict degree among the evidence bodies.

The conflict degree cannot be fully described only by conflict index *K* [12]. Hence, evidence distance [13] which represents the difference among evidence bodies is used jointly to completely describe the conflict degree of BPAs.

Assume that *d*_*ij*_ represents the evidence distance of BPAs *m*_*i*_ and *m*_*j*_; then, in this paper, *d*_*ij*_ can be expressed as

(3) $$\begin{array}{c} d_{ij}=\sqrt{{\left[m_{i}\left(A_{1}\right)-m_{j}\left(A_{1}\right)\right]}^{2}+{\left[m_{i}\left(A_{2}\right)-m_{j}\left(A_{2}\right)\right]}^{2}}. \end{array}$$

Then the comprehensive conflict index of *m*_*i*_ and *m*_*j*_ which is denoted by *C*_*ij*_ can be expressed in

(4) $$\begin{array}{c} C_{ij}=\frac{\left(C_{ij}^{\prime }+C_{ij}^{\prime \prime }\right)}{2}, \end{array}$$

where $C_{ij}^{\prime }=\sqrt{d_{ij}\times k_{ij}}$, *C*_*ij*_′′ = (*d*_*ij*_ + *k*_*ij*_)/2, and *k*_*ij*_ represents the conflict index of BPAs *m*_*i*_ and *m*_*j*_.

If all evidence bodies are compatible with each other, comprehensive conflict index *C*_*ij*_ is normally less than 0.5, and ideal results can be obtained with (2). If there is a high conflict degree among evidence bodies, *C*_*ij*_ will be greater than 0.5 due to the uncertainty of information. Under this condition, the results obtained with (2) will not be in accordance with reality.

In order to eliminate the impact of bad data on decision making and make the combination of BPAs to reflect the reality accurately, it is needed to improve the evidence theory. The evidence theory can be improved from the following two aspects: modification of original evidence body and modification of combination rule of BPAs [14].

#### 3.1.1. Modification of Original Evidence Body

In this paper, action states of traditional protections and direction components are taken as evidence bodies to lower the requirement of sampling synchronization.

The traditional primary protection based on double-terminal electrical quantities and direction comparison protection based on double-terminal direction components can identify the faulty line with certainty. Hence, they can be directly used as evidence bodies. However, the traditional backup protection, such as distance protection, is based on the setting cooperation of single-terminal electrical quantity information. Hence, it identifies a line whether or not in fault condition with uncertainty. Meanwhile, the action states of different zones of distance protection are not independent of each other. Hence, in order to meet the requirement of D-S evidence theory that different evidence bodies should be independent of each other, the evidence body of distance protection should be modified. For the proposed WABP, it takes zones 1, 2, and 3 distance protection as one evidence body.

In this context, the adopted evidence bodies are, respectively, traditional primary protection, direction comparison protection, and distance protection. The three relay protection elements have different implementation principles with each other. Meanwhile, it is known that the relay protection elements with different principles are independent of each other. Hence, the three evidence bodies are independent of each other, which can meet the requirement of D-S evidence theory.

#### 3.1.2. Modification of Combination Rule of BPAs

For the part with consistency, common combination rule expressed in (2) is used. For the part with conflict, the local conflict should be assigned among the conflicting focal elements. Finally, the improved combination rule is expressed in

(5) $$\begin{array}{c} m\left(A\right)=\{\begin{array}{cc} \underset{\cap A_{i}=A}{\sum }\;\underset{1\leq j\leq n}{\prod }m_{j}\left(A_{i}\right)+\Delta \varphi \left(A\right) & A\neq \emptyset ,\Theta \\ 0 & A=\emptyset ,\Theta , \end{array} \end{array}$$

where *n* is the number of evidence bodies, *A*_*i*_ is the conflicting focal element which can be *A*_1_ or *A*_2_, and Δ*φ*(*A*) is the conflicting information assigned to *A*_*i*_. Δ*φ*(*A*) can be expressed as

(6) $$\begin{array}{c} \Delta \varphi \left(A\right)=\omega \left(A\right)\times K^{\prime },\\ \omega \left(A\right)=\frac{\sum_{j=1}^{n}\lambda_{j}m_{j}\left(A\right)}{\sum_{i=1}^{2}\sum_{j=1}^{n}\lambda_{j}m_{j}\left(A\right)},\\ K^{\prime }=1-P\left(A_{1}\right)-P\left(A_{2}\right),\\ P\left(A_{1}\right)=\underset{\cap A_{i}=A_{1}}{\sum }\;\overset{\;}{\underset{1\leq j\leq n}{\prod }}m_{j}\left(A_{i}\right),\\ P\left(A_{2}\right)=\underset{\cap A_{i}=A_{2}}{\sum }\;\underset{1\leq j\leq n}{\prod }m_{j}\left(A_{i}\right), \end{array}$$

where *P*(*A*_1_) and *P*(*A*_2_) represent the information of nonconflict part of *n* evidence bodies, *K*′ is the information of conflict part, *ω*(*A*) is the weight factor of conflict assignment, and *λ*_*j*_ is the weight factor of each BPA.

*λ*_*j*_ depends on the conflict degree between evidence body *j* and other evidence bodies. Let *λ*_*j*_ = *C*_*ij*_; then an *n* × *n* matrix of conflict degree as shown in (7) can be built:

(7) $$\begin{array}{c} \text{Conf}={\left[\begin{array}{ccccc} 0 & C_{12} & C_{13} & \cdots & C_{1n}\\ C_{21} & 0 & C_{23} & \cdots & C_{2n}\\ \vdots & \vdots & \vdots & \vdots & \vdots \\ C_{n1} & C_{n2} & \cdots & C_{n3} & 0 \end{array}\right]}_{n\times n}. \end{array}$$

The total conflict degree between evidence body *j* and other evidence bodies is the sum of elements of *j*th row or *j*th column in the conflict degree matrix, as given in

(8) $$\begin{array}{c} \text{Con}{\text{f}}_{j}=\overset{n}{\underset{i=1}{\sum }}\text{Conf}\left(j,i\right). \end{array}$$

Hence, the total support degree of other *n* − 1 evidence bodies to evidence body *j* which is called “reliability degree” is

(9) $$\begin{array}{c} \text{Trus}{\text{t}}_{j}=\left(n-1\right)-\text{Con}{\text{f}}_{j}. \end{array}$$

Then *λ*_*j*_ can be obtained with the normalization of reliability degree:

(10) $$\begin{array}{c} \lambda_{j}=\frac{\text{Trus}{\text{t}}_{j}}{\sum_{i=1}^{n}\text{Trus}{\text{t}}_{i}}. \end{array}$$

### 3.2. Assignment Principle of BPA Values

The maximum protection scope of zone 3 distance protection is chosen to be the protection information domain of local line. It means that protection information domain includes the action states of traditional protections and direction components of the local line and action states of distance protections of the adjacent lines in the positive direction.

Because of the different protection scopes of different protections, the reaction capabilities of different protections on fault which occurs on the local line or adjacent line are different. Hence, it is needed to assign BPA values of different protections according to the respective protection scopes.

The protection scopes of primary protection and direction comparison protection are the whole line, but the primary protection and direction comparison protection cannot protect the adjacent lines. Hence, the action of primary protection or direction comparison protection represents the fact that a fault occurs on the local line. If the primary protection and direction comparison protection do not act, the local line is not in fault condition. Hence, the BPA values of primary protection and direction comparison protection are shown in Table 1.

Since distance protection has direction, only the BPA values of distance protection of the local line and the adjacent lines in the positive direction are assigned. The assignment of BPA values of distance protection is based on the action states of distance protection and the identification result of suspicious faulty lines.

There are four types of action states of distance protection normally. Action state *①*: zones 1, 2, and 3 all act. Action state *②*: zones 2 and 3 act, but zone 1 does not act. Action state *③*: zone 3 acts, but zones 1 and 2 do not act. Action state *④*: zones 1, 2, and 3 do not act. If other action states happen, it may be caused by human setting error or interference of communication system. Under these conditions, the evidence body of distance protection will not be adopted for the combination of evidence bodies.

**Figure 2:**
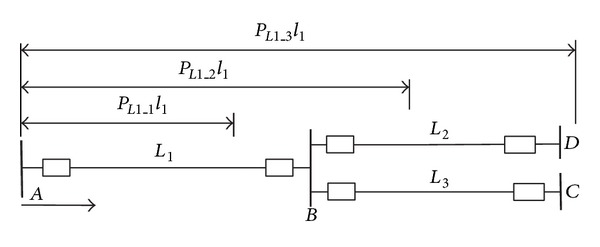
Protection area of zones 1, 2, and 3 distance protection.

Take the distance protection of *L*_1_ in Figure 2, for example, to elaborate the assignment principle of BPA values of distance protection.

In Figure 2, *P*_*L*1_1_, *P*_*L*1_2_, and *P*_*L*1_3_ are the normalized protection scopes of zones 1, 2, and 3 distance protection on *L*_1_, respectively. The lengths of *L*_1_, *L*_2_, and *L*_3_ are, respectively, denoted by *l*_1_, *l*_2_, and *l*_3_.

The assignment results of BPA values under different conditions are shown in Table 2. *x*, *y*_2_, and *y*_3_ represent the BPA values on fault condition of *L*_1_, *L*_2_, and *L*_3_, respectively.

In order to elaborate the assignment principle of BPA values of distance protection, the condition under which the action state *②* occurs and *L*_2_, *L*_3_ are both identified as suspicious faulty lines is taken for example. Firstly, under action state *②*, zone 2 distance protection of *L*_1_ acts, which means that the fault should occur in the protection scope of zone 2 distance protection of *L*_1_. Hence, the fault may occur on *L*_2_, *L*_3_ or the remote end of *L*_1_. However, *L*_1_ is not identified as suspicious faulty line, which means the fault does not occur on *L*_1_. As a consequence, *x* = 0.

For zone 2 distance protection of *L*_1_, its protection scope on *L*_2_ or *L*_3_ is (*P*_*L*1_2_ − 1)*l*_1_ without consideration of the influence of infeed current. Assume that, wherever the fault position is, the fault occurrence probability is the same. Hence, the occurrence probability of fault on *L*_2_ and within the protection scope of zone 2 distance protection of *L*_1_ is (*P*_*L*1_2_ − 1)*l*_1_/*l*_2_, and the occurrence probability of fault on *L*_3_ and within the protection scope of zone 2 distance protection of *L*_1_ is (*P*_*L*1_2_ − 1)*l*_1_/*l*_3_. With normalization, it can be obtained that

(11) y2=(PL1_2−1)l1/l2(PL1_2−1)l1/l2+(PL1_2−1)l1/l3=1/l21/l2+1/l3=l3l2+l3.

Likewise, *y*_3_ = *l*_2_/(*l*_2_ + *l*_3_).

### 3.3. Special Treatment under Condition of DC Power Supply Failure

If the DC power supply in substation is lost, the corresponding substation cannot upload information to master substation of WABP and its breakers cannot trip to clear the fault. Under this condition, WABP needs to identify whether fault occurs in local protection area of the substation whose DC power supply is lost. If a fault occurs in the local protection area, WABP should order the protections of adjacent substations to trip to clear the fault as remote backup protection.

Under condition of DC power supply failure, evidence bodies and assignment of BPA values should be modified to ensure the WABP can identify the faulted area. Taking the power grid shown in Figure 2, for example, assume that the DC power supply in substation *B* is lost and a fault occurs on *L*_1_. For *L*_1_, *L*_2_, and *L*_3_, their primary protections and direction comparison protections are in fault condition because of information loss. Hence, the action states of corresponding protections should not be adopted as evidence bodies. Only distance protection of *L*_1_ in substation *A*, distance protection of *L*_2_ in substation *C*, and distance protection of *L*_3_ in substation *D* can be adopted. Let *p* represent BPA value of distance protection on fault condition of the area. Under action states *①* and *②*, *p* = 1. Under action state *③*, as the sensitivity of zone 3 distance protection is not less than 1.2 when a fault occurs at the terminal of adjacent line, *p* = 1/1.2. Under action state *④*, *p* = 0.

## 4. Implementation of the Proposed WABP 

### 4.1. Basic Implementation Principle

**Figure 3:**
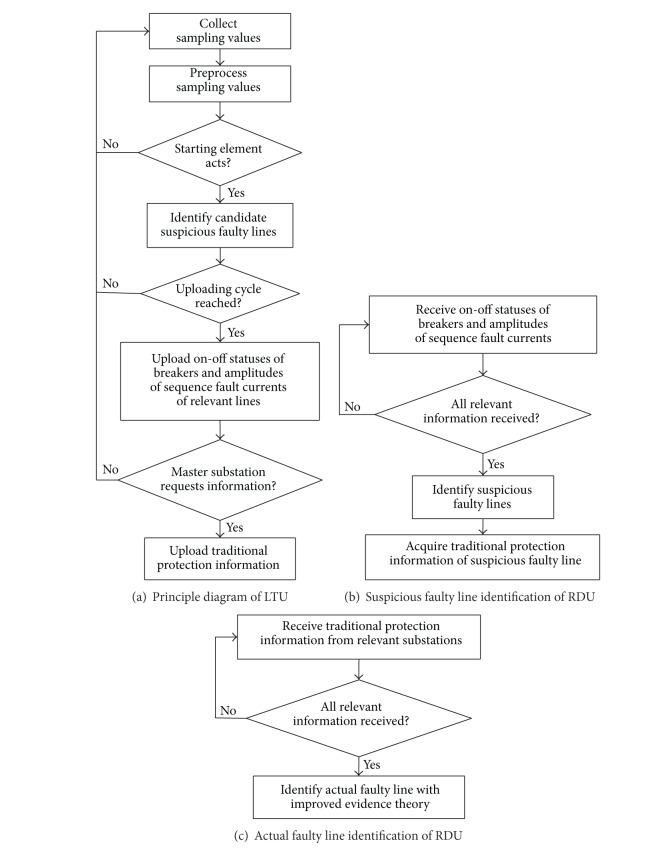
Fundamental principle diagram of the proposed WABP.

Assume that intelligent electronic device (IED) of the proposed WABP is installed in each slave substation and master substation. The IED in slave substation is defined as local terminal unit (LTU), and IED in master substation is defined as region decision unit (RDU). The basic principle of the proposed WABP is shown in Figure 3.

1. First, LTU collects the sampling values of electrical quantities of each transmission line in the local substation, and it will preprocess the sampling values to improve the reliability of measurement information. Then LTU detects the action state of starting element of each line. If at least one of the starting elements acts, LTU will identify the candidate suspicious faulty lines which are the closest to the fault point. After that, LTU will upload the on-off statuses of breakers and amplitudes of sequence fault currents of the candidate suspicious faulty lines to RDU.
2. RDU receives in real time the uploaded information. After all the information has been received, the on-off statuses of breakers are used to identify the structure of power grid, and the amplitudes of sequence fault currents are used to identify the suspicious faulty lines. Then RDU asks the relevant substations within the protection information domain of suspicious faulty lines to upload the action states of traditional protections and direction components.
3. Relevant substations transmit the action states of traditional protections and direction components to RDU. After all the relevant protection information has been received completely, master substation identifies the actual faulty line based on the improved evidence theory.

### 4.2. Identification of Candidate Suspicious Faulty Line

In order to reduce the wide-area communication flow, the following identification method of candidate suspicious faulty lines is proposed.

(1) LTU firstly detects the action state of starting element of each line in the local substation, to judge whether a fault occurs.

For asymmetrical fault, starting criteria are expressed in

(12) $$\begin{array}{c} \left(\frac{V_{L0}}{V_{N}}\geq K_{\text{ZV}}\right)U\left(\frac{I_{L0}}{I_{N}}\geq K_{\text{ZI}}\right) \end{array}$$

or

(13) $$\begin{array}{c} \left(\frac{V_{L2}}{V_{N}}\geq K_{\text{NV}}\right)U\left(\frac{I_{L2}}{I_{N}}\geq K_{\text{NI}}\right), \end{array}$$

where *V*_*L*0_, *V*_*L*2_, *V*_*N*_, *I*_*L*0_, *I*_*L*2_, and *I*_*N*_ are the amplitudes of zero-sequence voltage, negative-sequence voltage, rated voltage, zero-sequence current, negative-sequence current, and rated current of line *L*, respectively. *K*_ZV_, *K*_NV_, *K*_ZI_, and *K*_NI_ are the scale factors of zero-sequence voltage, negative-sequence voltage, zero-sequence current, and negative-sequence current, respectively.

According to the typical setting value of common pick-up element, the four scale factors can be set as 0.1 to ensure the sensitivity for detecting the grounded fault via high resistance and other kinds of complex fault. Meanwhile, setting the four scale factors to be 0.1 can avoid the maloperation of the pick-up element caused by the measuring error of CT (smaller than 10%).

The starting criterion for symmetrical fault is given in

(14) $$\begin{array}{c} \frac{V_{L1}}{V_{N}}\leq K_{\text{PV}}, \end{array}$$

where *V*_*L*1_ is the amplitude of positive-sequence voltage of line *L* and *K*_PV_ is the scale factor of positive-sequence voltage.

Under symmetrical fault condition, the short-circuit current is rather large, which causes the positive-sequence voltage to drop greatly. In this paper, *K*_PV_ is set as 0.5, to reduce the number of nonfaulty lines whose pick-up elements trip and lower the wide-area communication flow.

(2) If there are transmission lines whose start elements act when a fault occurs, LTU will compare the amplitudes of sequence fault currents of corresponding lines. The transmission line with the maximum sequence fault current should be identified as candidate suspicious faulty line. However, in order to ensure the reliability, the error of CT should be taken into consideration. Since the error of electronic current transformer in smart substation should meet the requirement of 5P or 5TPE level, the maximum error between two current transformers is 10%. Hence, the transmission line whose amplitude of sequence fault current is not smaller than 90% of the maximum amplitude should also be identified as candidate suspicious faulty line.

Therefore, the identification principle of candidate suspicious faulty line is summarized as follows. If (12) is satisfied, the transmission line whose amplitude of zero-sequence fault current is the largest or not smaller than 90% of the maximum amplitude is identified as the candidate suspicious faulty line. If (13) is satisfied, the transmission line whose amplitude of negative-sequence fault current is the largest or not smaller than 90% of the maximum amplitude is identified as the candidate suspicious faulty line. If (14) is satisfied, the transmission line whose amplitude of fault current is the largest or not smaller than 90% of the maximum amplitude is identified as the candidate suspicious faulty line.

### 4.3. Decision Making of the Actual Faulty Line

After the information uploaded by relevant substations has been received completely, the decision-making center of master substation takes the following steps to identify the actual faulty line.

(1) The amplitudes of sequence fault currents uploaded by relevant substations will be sorted in descending order. The transmission line whose amplitude of sequence fault current is the largest or not smaller than 90% of the maximum amplitude is identified as the suspicious faulty line. If amplitudes of one terminal of a transmission line are lost, the transmission line is also regarded as the suspicious faulty line.

(2) The decision-making center gets logic quantity information of the identified suspicious faulty lines from relevant substations and identifies the actual faulty line with the improved evidence theory.

Take the condition that a fault occurs on *L*_3_ in Figure 1, for example, to elaborate the identification method of the actual faulty line. Assuming the identified suspicious faulty lines are *L*_1_ and *L*_3_, the decision-making steps are stated as follows.

(i) Determine the BPA value of each evidence body of *L*_3_ based on the action states of traditional protections and direction components within the protection information domain of *L*_3_.

(ii) Identify conflict index of evidence bodies of *L*_3_ according to (4). If *C*_*ij*_ < 0.5, the comprehensive BPA value can be obtained according to (1). Otherwise, (2) is used to get the comprehensive BPA value. Similarly, the comprehensive BPA value of *L*_1_ can be obtained.

(iii) The protection scope is so large that the mal-operation of WABP will possibly make many transmission lines out of service by mistake. Hence, the reliability requirement of the decision making of WABP is extremely strict. In order to improve the reliability of decision making, the following decision-making strategy is proposed. If the comprehensive BPA value is in [0, 1/3) which is called “certain nonfault section,” the line is identified in normal operating condition. If the comprehensive BPA value is in [1/3, 2/3] which is called “uncertain section,” the operating condition of line cannot be identified. If the comprehensive BPA value is in (2/3, 1] which is called “certain fault section,” the line is identified in fault condition. The uncertain section can help avoid the appearance of wrong decision on condition that the conflict of evidence bodies is large.

Therefore, if *m*_*L*1_(*A*_2_) and *m*_*L*3_(*A*_2_) are both in the certain fault section, compare their values. The line with larger BPA value is identified as the faulty line. Otherwise, the decision-making result of the section which *m*_*L*1_(*A*_2_) or *m*_*L*3_(*A*_2_) falls in is the identification result of the operating condition of the corresponding line.

The occurrence possibility of multiple faults is small. Under the condition of multiple faults, the possibility that primary protections at multiple points all fail to trip and the multiple faults need to be cleared by WABP is even much smaller. Hence, only single fault is taken into account in this paper.

## 5. Simulation Example

**Figure 4:**
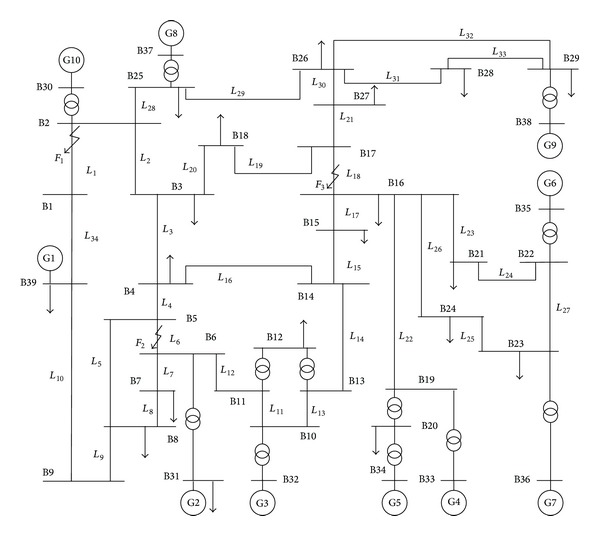
Model of IEEE 10-generator-39-bus system.

In order to validate the performance of the proposed WABP, simulation model of IEEE 10-generator-39-bus is built with PSCAD/EMTDC, as shown in Figure 4. The parameters of generators, transformers, and transmission lines in [15] are used here.

Lots of simulation cases have been studied to validate the performance of the proposed WABP. The following three fault conditions are chosen to be the representative fault conditions.

Fault condition *①*: a fault occurs at point *F*_1_ which locates at 20% of the full length away from B1 of *L*_1_. Fault condition *②*: a fault occurs at point *F*_2_ which locates at 50% of the full length of *L*_6_. Fault condition *③*: a fault occurs at point *F*_3_ which locates at 10% of the full length away from B16 of *L*_18_.

Besides, the simulations are divided into two parts: *①* performance test of the identification of suspicious faulty line; *②* fault tolerance performance test of the proposed WABP.

### 5.1. Performance of the Suspicious Faulty Line Identification

In order to validate the performance of the suspicious faulty line identification, the tested fault types include single-phase-grounded fault, single-phase-grounded fault via high resistance, phase-to-phase fault, phase-to-phase fault via transition resistance, phase-to-phase-grounded fault, and three-phase fault.

Table 3 shows the simulation results of the suspicious faulty line identification under different fault conditions.

The “68” in Table 3 indicates that there are 68 relaying protection locations in IEEE 10-generator-39-bus system since there are 34 transmission lines. No matter which kind of fault occurs, the number of uploaded amplitudes of sequence fault currents is much less than the total number of protection locations, as shown in Table 3. Especially, under condition of single-phase-grounded fault via high resistance, the number of uploaded amplitudes is greatly reduced. If a symmetrical fault occurs, the number of uploaded amplitudes is limited as low voltage starting criterion is adopted. Hence, the proposed WABP can greatly reduce the wide-area communication flow.

As shown in Table 3, the proposed identification method of suspicious faulty line can accurately and uniquely identify the actual faulty line under normal situations. Only in a few cases, such as series lines *L*_1_ and *L*_34_, there are two suspicious faulty lines. Hence, the effectiveness of the suspicious faulty line identification method is verified. Meanwhile, the processing burden of the decision-making center and wide-area communication flow are further reduced because only a few candidate suspicious faulty lines are identified.

### 5.2. Fault Tolerance Performance of the Proposed WABP

In order to validate the fault tolerance performance of the proposed WABP, the following three conditions are taken into consideration: *①* logic quantity information is mistaken; *②* logic quantity information is lost; *③* DC power supply is lost.

#### 5.2.1. Performance of the Proposed WABP When Logic Quantity Information Is Mistaken

The following two mistaken conditions of logic quantity information are taken into account to validate the performance of the proposed WABP. Mistaken condition *①*: there are 1 to 10 random mistakes of logical quantity information. Mistaken condition *②*: 1 to 5 protection devices are in fault condition, and all the logic quantity information of corresponding protection devices is mistaken.

For the fault which occurs on *L*_1_, the simulation results under mistaken condition *①* are shown in Table 4, and the simulation results under mistaken condition *②* are shown in Table 5.

For the fault which occurs on *L*_6_ or *L*_18_, the simulation results under mistaken condition *①* are given in Table 6, and the simulation results under mistaken condition *②* are given in Table 7.

(i) For the fault which occurs on *L*_1_, as shown in Table 4, on condition that 1 to 6 evidence bodies are mistaken, the correct decision-making result can be obtained to identify *L*_1_ as faulty line; on condition that 7 or 8 evidence bodies are mistaken, the comprehensive BPA values fall into the uncertain section, the certain decision-making result cannot be obtained, and the proposed WABP will be out of service in these three conditions; on condition that more than 8 evidence bodies are mistaken, the wrong result will be obtained.

(ii) Let *N*_*m*_ represent the number of protection devices whose logic quantity information is all mistaken. For the fault which occurs on *L*_1_, as shown in Table 5, the correct decision-making result can be obtained to identify *L*_1_ as faulty line when *N*_*m*_ is less than 3; under other conditions, the incorrect decision-making result will be obtained.

(iii) For the fault which occurs on *L*_6_, as shown in Table 6, on condition that 1 to 7 evidence bodies are mistaken, the correct decision-making result can be obtained to identify *L*_6_ as faulty line; on condition that 8 or 9 evidence bodies are mistaken, the certain decision-making result cannot be obtained and the proposed WABP will be out of service in these three conditions; on condition that more than 10 evidence bodies are mistaken, the wrong decision-making result will be obtained.

For the fault which occurs on *L*_18_, as shown in Table 6, on condition that 1 to 8 evidence bodies are mistaken, the correct decision-making result can be obtained to identify *L*_18_ as faulty line; under other conditions, the correct decision-making result cannot be obtained.

(iv) For the fault which occurs on *L*_6_, as shown in Table 7, the correct decision-making result can be obtained to identify *L*_6_ as faulty line when *N*_*m*_ is less than 4; under other conditions, the correct decision-making result cannot be obtained.

For the fault which occurs on *L*_18_, as shown in Table 7, the correct decision-making result can be obtained to identify *L*_18_ as faulty line when *N*_*m*_ is less than 5; under other conditions, the correct decision-making result cannot be obtained.

#### 5.2.2. Performance of the Proposed WABP When Logic Quantity Information Is Lost

In order to validate the performance of the proposed WABP when logic quantity information is lost, logic quantity information of a whole protection device is considered as a unit.

For the fault which occurs on *L*_1_, the simulation results are given in Table 8. For the fault which occurs on *L*_6_ or *L*_18_, the simulation results are given in Table 9.

Let *N*_*l*_ represent the number of protection devices whose logic quantity information is all lost. For the fault which occurs on *L*_1_, as Table 8 shows, for the fault which occurs on *L*_1_, the correct decision-making result can be obtained to identify *L*_1_ as faulty line when *N*_*l*_ is less than 6; when *N*_*l*_ is up to 6~8, the certain decision-making result cannot be obtained and the proposed WABP will be out of service in these three conditions; when *N*_*l*_ is larger than 8, the wrong decision-making result will be obtained.

For the fault which occurs on *L*_6_, as Table 9 shows, the correct decision-making result can be got to identify *L*_6_ as faulty line when *N*_*l*_ is less than 6; when *N*_*l*_ is up to 6~10, the certain decision-making result cannot be obtained and the proposed WABP will be out of service in these three conditions; when *N*_*l*_ is larger than 10, the wrong decision-making result will be obtained.

For the fault which occurs on *L*_18_, as shown in Table 9, the correct decision-making result can be obtained to identify *L*_18_ as faulty line even when *N*_*l*_ is up to 11, since the number of branches connected with B16 or B17 is large.

Actually, the reliabilities of protection devices and communication network are high enough under usual condition. The possibility that logical quantity information of multiple protection devices is all lost simultaneously is small. Hence, the reliability of the proposed WABP is rather high under usual conditions.

#### 5.2.3. Performance of the Proposed WABP under Condition of DC Power Supply Failure

When a fault occurs in the local protection area of the substation whose DC power supply is lost, the identification results of faulted area are shown in Table 10.

It can be obtained from Table 10 that, when a fault occurs in the local protection area of the substation whose DC power supply is lost, the proposed WABP can identify the faulted area correctly.

From the above simulation results, it can be obtained that the proposed WABP has excellent performance even on condition that logic quantity information is mistaken or lost, or DC power supply is lost. It means that the proposed WABP has high fault tolerance and reliability.

## 6. Conclusion

To solve the problems of the existing WABP algorithms, such as high requirement of sampling synchronization, large amount of wide-area communication flow, and low fault tolerance, a novel WABP algorithm based on the distribution characteristics of fault component current and improved evidence theory is proposed.

As only logical quantity information and amplitudes of electrical quantities are uploaded, the proposed WABP has low requirement of sampling synchronization. The proposed WABP identifies suspicious faulty line based on the distribution characteristics of fault component current, and only information of a few transmission lines is uploaded. Hence, the wide-area communication flow and processing burden of the decision-making center of RTU are reduced greatly. Meanwhile, improved evidence theory based on the action states of traditional protections and direction components is applied. Hence, the novel WABP algorithm has high fault tolerance. The features of the proposed WABP make it suitable for practical application.

## Figures and Tables

**Table 1:** BPA assignment of primary protection and direction comparison protection.

Action state	BPA value of local line	
	Normal	Fault
Act	0	1
No act	1	0

**Table 2:** Assignment results of BPA values of distance protection of *L*_1_.

Suspicious faulty line	Action state			
	①	②	③	④
*L*_1_	*x* = 1, *y*_2_ = *y*_3_ = 0	*x* = 1, *y*_2_ = *y*_3_ = 0	*x* = *y*_2_ = *y*_3_ = 0	*x* = *y*_2_ = *y*_3_ = 0

*L*_2_ and *L*_3_	*x* = 0, *y*_2_ = *l*_3_/(*l*_2_ + *l*_3_), *y*_3_ = *l*_2_/(*l*_2_ + *l*_3_)	*x* = 0, *y*_2_ = *l*_3_/(*l*_2_ + *l*_3_), *y*_3_ = *l*_2_/(*l*_2_ + *l*_3_)	*x* = 0, *y*_2_ = *y*_3_ = 0.5	*x* = *y*_2_ = *y*_3_ = 0

*L*_2_	*x* = 0, *y*_2_ = 1, *y*_3_ = 0	*x* = 0, *y*_2_ = 1, *y*_3_ = 0	*x* = 0, *y*_2_ = 1, *y*_3_ = 0	*x* = *y*_2_ = *y*_3_ = 0

*L*_3_	*x* = 0, *y*_2_ = 0, *y*_3_ = 1	*x* = 0, *y*_2_ = 0, *y*_3_ = 1	*x* = 0, *y*_2_ = 0, *y*_3_ = 1	*x* = *y*_2_ = *y*_3_ = 0

*L*_1_ and *L*_2_	*x* = 1, *y*_2_ = *y*_3_ = 0	*x* = (1 − *P*_*L*1_1_)/(*P*_*L*1_2_ − *P*_*L*1_1_), *y*_2_ = (*P*_*L*1_2_ − 1)/(*P*_*L*1_2_ − *P*_*L*1_1_), *y*_3_ = 0	*x* = 0, *y*_2_ = 1, *y*_3_ = 0	*x* = *y*_2_ = *y*_3_ = 0

*L*_1_ and *L*_3_	*x* = 1, *y*_2_ = *y*_3_ = 0	*x* = (1 − *P*_*L*1_1_)/(*P*_*L*1_2_ − *P*_*L*1_1_), *y*_2_ = 0, *y*_3_ = (*P*_*L*1_2_ − 1)/(*P*_*L*1_2_ − *P*_*L*1_1_)	*x* = 0, *y*_2_ = 0, *y*_3_ = 1	*x* = *y*_2_ = *y*_3_ = 0

**Table 3:** The number of uploaded amplitudes of sequence fault currents and identified suspicious faulty line.

Fault types	Number of uploaded amplitudes of sequence fault currents/total number of protection locations/suspicious faulty line		
	*F*_1_	*F*_2_	*F*_3_
AG	5/68/*L*_1_, *L*_34_	5/68/*L*_6_	14/68/*L*_18_
BC	6/68/*L*_1_, *L*_34_	14/68/*L*_6_	23/68/*L*_18_
BCG	5/68/*L*_1_, *L*_34_	12/68/*L*_6_	18/68/*L*_18_
ABC	3/68/*L*_1_, *L*_34_	5/68/*L*_6_	7/68/*L*_18_
BC (25 Ω)	5/68/*L*_1_, *L*_34_	13/68/*L*_6_	16/68/*L*_18_
AG (200 Ω)	3/68/*L*_1_, *L*_34_	4/68/*L*_6_	2/68/*L*_18_

**Table 4:** WABP performance under mistaken condition ① when a fault occurs on *L*_1_.

Comprehensive BPA values on fault condition	Number of mistaken evidence bodies									
	1	2	3	4	5	6	7	8	9	10
*L*_1_	0.986	0.955	0.928	0.887	0.818	0.706	0.571	0.429	0.294	0.182
*L*_34_	0.017	0.053	0.091	0.148	0.263	0.417	0.583	0.737	0.857	0.938

**Table 5:** WABP performance under mistaken condition ② when a fault occurs on *L*_1_.

Comprehensive BPA value on fault condition	Number of protection devices whose logic quantity information is all mistaken				
	1	2	3	4	5
*L*_1_	0.926	0.728	0.494	0.263	0.176
*L*_34_	0.307	0.568	0.741	0.857	0.905

**Table 6:** WABP performance under mistaken condition ① when a fault occurs on *L*_6_ or *L*_18_.

Faulty line	Number of mistaken evidence bodies									
	1	2	3	4	5	6	7	8	9	10
*L*_6_	1.000	0.991	0.968	0.929	0.868	0.786	0.682	0.563	0.438	0.318
*L*_18_	1.000	0.994	0.981	0.958	0.923	0.875	0.813	0.736	0.647	0.55

**Table 7:** WABP performance under mistaken condition ② when a fault occurs on *L*_6_ or *L*_18_.

Faulty line	Number of protection devices whose logic quantity information is all mistaken				
	1	2	3	4	5
*L*_6_	0.991	0.868	0.682	0.438	0.318
*L*_18_	0.994	0.923	0.813	0.736	0.647

**Table 8:** WABP performance under condition of information loss when a fault occurs on *L*_1_.

Comprehensive BPA values on fault condition	Number of protection devices whose logic quantity information is all lost								
	1	2	3	4	5	6	7	8	9
*L*_1_	0.903	0.872	0.784	0.743	0.688	0.615	0.5	0.413	0.259
*L*_34_	0.116	0.128	0.143	0.185	0.257	0.278	0.222	0222	0.222

**Table 9:** WABP performance under condition of information loss when a fault occurs on *L*_6_ or *L*_18_.

Faulty line	Number of protection devices whose logic quantity information is all lost										
	1	2	3	4	5	6	7	8	9	10	11
*L*_6_	1.00	0.75	0.73	0.72	0.68	0.63	0.57	0.54	0.50	0.41	0.26
*L*_18_	1.00	0.82	0.78	0.76	0.74	0.71	0.68	0.69	0.70	0.71	0.72

**Table 10:** WABP performance under condition of DC power supply failure.

Substation with DC power supply failure	Faulty line	Comprehensive BPA value	Identified faulted area
B1	*L*_1_	0.9166	*L*_1_, *L*_34_
B16	*L*_18_	0.8729	*L*_17_, *L*_18_, *L*_22_, *L*_23_, *L*_26_
